# Medical disinformation and the unviable nature of COVID-19 conspiracy theories

**DOI:** 10.1371/journal.pone.0245900

**Published:** 2021-03-12

**Authors:** David Robert Grimes

**Affiliations:** 1 School of Physical Sciences, Dublin City University, Dublin, Leinster, Ireland; 2 Department of Oncology, University of Oxford, Roosevelt Drive, Oxford, Oxfordshire, United Kingdom; University Magna Graecia of Catanzaro, ITALY

## Abstract

The coronavirus pandemic has seen a marked rise in medical disinformation across social media. A variety of claims have garnered considerable traction, including the assertion that COVID is a hoax or deliberately manufactured, that 5G frequency radiation causes coronavirus, and that the pandemic is a ruse by big pharmaceutical companies to profiteer off a vaccine. An estimated 30% of some populations subscribe some form of COVID medico-scientific conspiracy narratives, with detrimental impacts for themselves and others. Consequently, exposing the lack of veracity of these claims is of considerable importance. Previous work has demonstrated that historical medical and scientific conspiracies are highly unlikely to be sustainable. In this article, an expanded model for a hypothetical *en masse* COVID conspiracy is derived. Analysis suggests that even under ideal circumstances for conspirators, commonly encountered conspiratorial claims are highly unlikely to endure, and would quickly be exposed. This work also explores the spectrum of medico-scientific acceptance, motivations behind propagation of falsehoods, and the urgent need for the medical and scientific community to anticipate and counter the emergence of falsehoods.

## Introduction

Conspiracy theories about aspects of medicine have long existed, positing that sinister motivations underpin everything from vaccination campaigns to cancer treatment [1–7]. While this has been a problem since before the dawn of social media, it has been hugely exacerbated by the dubious amplification [8] that social media provides. The scope of this problem is hard to overstate—in one study of over 126,000 news stories discussed online, researchers found that by virtually any metric falsehoods, rumours, and hoaxes far eclipsed more trustworthy information [9]. The COVID-19 pandemic has seen this propensity for falsehood raise to dizzying heights. Such is the abundance of misinformation (born of misconception) and disinformation (deliberate fictions) that the World Health Organisation note we face a shadow problem alongside the ongoing pandemic—an infodemic, the ‘..overabundance of information, some accurate and some not, that makes it hard for people to find trustworthy sources and reliable guidance when they need it’.

Even before COVID-19, the consequence of this on public acceptance of medical science has been alarming. Exposure to anti-vaccine propaganda, for example, is a major driver of vaccine hesitancy, heavily influencing parental intentions to vaccinate [4]. Such is the dominance of anti-vaccine disinformation online that is has seriously diminished vaccine uptake worldwide, leading to a dark renaissance of once-virtually conquered diseases. Consequently, the WHO declared “vaccine hesitancy” a top ten threat to health in 2019. In fields as critical as oncology, alternative medicine advocates frequently denounce interventions such as radiotherapy and chemotherapy as “poisons” to profit pharmaceutical companies, or insist they know of a cancer cure suppressed by main-steam medicine. Consequences for patients sucked into these narratives can be life-limiting and harmful [8, 10].

### Susceptibility to medico-scientific conspiracy theory

**Fig 1:**
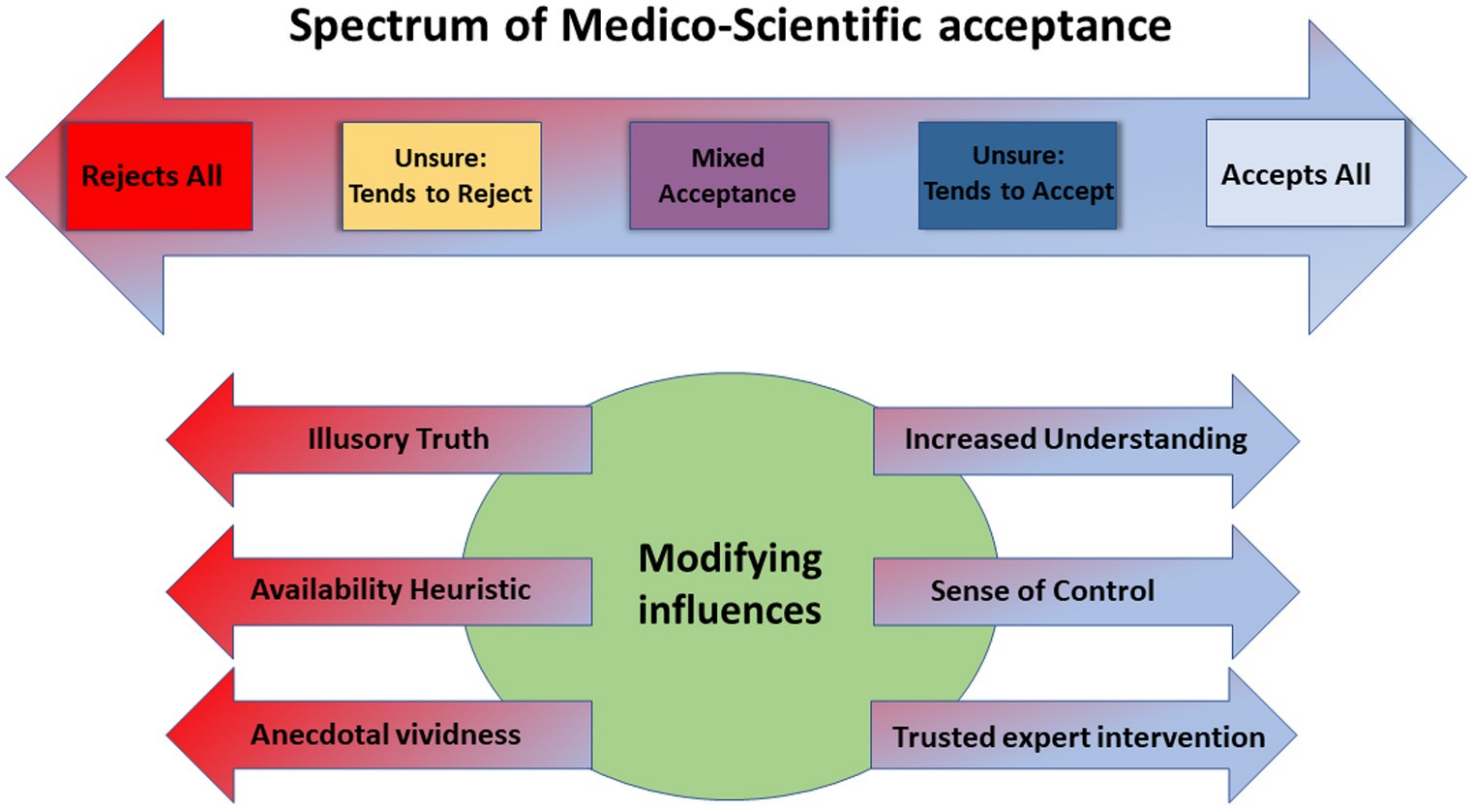
The spectrum of medico-scientific acceptance.

It is easy but misguided to dismiss this as the domain of a small cohort of unreachable cranks, but this is to underplay the problem. Mere exposure to medical myths can distort the perception of unsuspecting individuals, to our collective detriment. Acceptance of medico-scientific consensus and guidance is likely not a simple binary; in studies of vaccine up-take, vaccine hesitancy exists on a spectrum, which can be readily influenced by several mechanisms [11–13]. In this paper, we posit a similar continuum for acceptance of medico-scientific consensus, as outlined in Fig 1.

The precise gradient and distribution of this spectrum is highly likely to vary by country, specific topic, and background. In studies of vaccine hesitancy, for example, the 2020 Vaccine confidence project report [14] in the EU and UK found that over-all, 50% of respondents agreed strongly with the Statement “Vaccines are Safe”, with a further 37% tending to agree, 3% unsure, 6% tending to disagree and 3% strongly disagreeing. This figure, however, varied even for different vaccinations and more markedly across countries; in Portugal, 70% of strongly agreed with the statement that vaccines were safe, with only 36% of respondents in Hungary agreeing. In relation to a COVID-19 vaccine specifically, similar variation is seen worldwide, with over 90% of Chinese respondents agreeing they would take such a vaccine, while only 55% of those in Russia concurred [15].

There are also a range of influences which literature suggests can act to increase rejection of medical science. A non-exhaustive list of these factors would include

1. **Illusory truth phenomenon**: This is the observation that repeated exposure to falsehood can prime us to implicitly accept it, even when we are aware on an intellectual level that it is false [16–18]. When disinformation on health dominates, it is likely this would act to drive rejection of medico-scientific standpoints.
2. **The availability heuristic**: Our current evidence base suggests we afford more weight to more readily recalled information, even when this might be misleading [19, 20]. Medico-scientific conspiracy claims are typically shocking, which makes more more easily recalled, and increases their apparent importance.
3. **Fallacy of anecdotal vividness**: We tend to react more viscerally to emotive claims than more sober-headed analysis. Accordingly, alarming yet unsubstantiated health claims frequently garner undue traction, typically when coupled with an ostensibly personal account [21, 22].

These influences can be extraordinarily difficult to counter, and there is only limited data on what interventions might work to reduce conspiratorial ideation and acceptance of bogus narratives. Some effective methods might include

1. **Increasing understanding**: Epistemic uncertainty tends to drive conspiratorial thinking [23], and there is evidence that this can be counteracted by improving understanding, rendering them less likely to embrace pseudo-scientific narratives [24].
2. **Instilling a sense of control**: Acceptance of conspiracy theory may be a reaction to a diminished sense of control in one’s circumstances, even if those circumstances are not directly related to the subject of the conspiracy [25, 26]). While the precise impact of control remains unclear [27], it is possible that some acceptance of conspiracy theory might be mitigated by interventions or actions that improve one’s general sense of agency.
3. **Expert intervention**: The influence of trusted experts can also help improve acceptance of medical science. A physicians recommendation to vaccinate, for example, has marked effect on parental decisions to [28–30].

### COVID-19 conspiracy theories

COVID-19 has seen a plethora of conspiracy theories adopted worldwide, specific to the pandemic, which has been propagated heavily across social media. Much of this is organic, arising from already existent conspiracy theories. An EU commission report, however, found ample evidence that Russian and Chinese state forces in particular had amplified and propagated conspiracy theories about COVID-19 [31], a finding echoed in American intelligence reports [32]. Such disinformation is typically spread with the aim of undermining societal cohesion in rival nations and sowing seeds of mistrust. Whether organic conspiracy theory or disinformation campaign, a non-exhaustive list of common themes include the assertions that

- **A. COVID-19 is a hoax / deliberately engineered**: Since the dawn of the pandemic, a dichotomous set of narratives either dismissing the novel coronavirus as an outright hoax or alternatively insisting it has been engineered and spread have garnered serious traction. Typically, these claims posit that the virus, either fictional or engineered, is a means to suppress freedoms on a global scale. While such narratives seem mutually opposed, they are frequently held in tandem by a cohort of believers despite mutual exclusivity—a not infrequent situation with conspiratorial thinking [33].
- **B. COVID-19 is a pretext for a mass vaccination programme**: The idea that COVID-19 is a pretence for a campaign of mandatory vaccination has been been unduly popular, amplified by anti-vaccine figures with considerable uptake. Many of these claims focus on philanthropist Bill Gates, who it is claimed is using the pandemic as a means to microchip people with vaccines.
- **C. COVID-19 is caused by 5G electromagnetic radiation**: One of the most bizarre narratives around COVID-19 is the idea is is caused by 5G radiation. Such is the depth of feeling on this issue that it has led to a spate of arson attacks on cell-phone towers the world over. 5G radiation is, however, neither ionizing nor capable of inducing a virus, a supposition which appears a biological impossibility. Prior to the advent of the novel coronavirus, opposition to 5G had already been firmly established worldwide, reciting long debunked myths [34]. The erroneous link to COVID-19, however, has been perpetuated by celebrities and has become an extremely enduring claim by fringe groups throughout the course of the pandemic.

Outlandish as these claims might seem, they hold considerable sway. 25% of Americans surveyed said it was definite or probable that COVID-19 was planned, a figure which raised to 48% for those with a high-school education or less [35]. Another American study in July 2020 found that 37% of respondents believed COVID-19 to be a bio-weapon deliberately engineered by the Chinese [36]. Nor is this solely an American phenomena: in the UK, 30% of those surveyed deemed the statement “COVID-19 was probably created in a lab” to be true [37]. Similarly, claims that the pandemic is a ruse by pharmaceutical companies to force vaccination on people have ample international resonance, with 13% and 17% [36] in the UK and USA respectively agreeing with this sentiment. Beliefs 5G causes COVID-19 are also prevalent worldwide, with 8% of UK respondents agreeing COVID is caused by 5G [37], a position echoed by 12% of Australians [38].

In addition to these major strands, other ideas which have established a foot-hold include claims that face-masks can lead to acute hypoxia and carbon dioxide poisoning, which while completely false has fuelled anti-mask protests the world over. These have drawn considerable crowds, such as the convergence of over 17,000 protesters in Berlin in August 2020. Others claim the virus is no worse than seasonal influenza, despite the abundance of evidence to the contrary. These claims are harmful not only to adherents, but to those around them, as evidence to date suggests subscribers to these narratives do not take precautionary infection control measures, putting others at risk [39, 40].

### Motivation for this work

Given the sheer prevalence of these claims worldwide, it is absolutely imperative they be thoroughly debunked. Physicians and scientists are at the forefront of efforts to highlight the lack of veracity and danger of these commonly held beliefs. To mitigate their influence, however, effective strategies are crucial. Merely imparting information is rarely enough to sway individuals, however, and for those further on the medico-scientific acceptance spectrum, an information-deficit approach can sometimes paradoxically backfire [41]. This may be even more likely with COVID and medical conspiracy theories in general, because affected individuals might feel powerless, and that their concerns are not heard.

Consequently, a more Socratic approach might be more effective at disabusing people of such misconceptions. Instead of dismissing concerns, we can ask questions of adherents which stimulates critical thinking, giving scope for the changing of views. In a previous investigation of those who believed the Moon-landing to be a hoax, it was found that a Socratic approach where their beliefs were critically assessed reduced the acceptance of pseudo-scientific narratives [24]. In previous work by the author, a Devil’s advocate approach was outlined regarding several scientific conspiracy theories, regarding the moon-landings, vaccination, climate-change, and hidden cancer cures [42]. Instead of dismissing these outright, it was initially assumed such conspiracies existed, and a simple mathematical model was outlined to consider the ramifications of this. In this work, it was shown that even under ideal circumstances for conspirators, the sheer scale of the enterprise rendered them unviable for any considerable period of time.

These previous conspiratorial narratives were long established, but COVID remains an evolving situation with increasingly outlandish narratives arising around it. We accordingly take a similar but modified approach to counteracting these claims. This approach considers estimates specific to COVID-19, and unveils an updated model accounting for the existing dynamic scenario, to quantify how vanishingly unlikely COVID medico-scientific conspiracies are to endure under even ideal circumstances.

## Materials and methods

### Modelling conspiratorial viability

Previous work by the author [42, 43] has modelled the viability of any hypothetical conspiracy by assuming that conspirators operate to conceal their undertakings. A leak of information is sufficient to expose a conspiracy, and this can be either accidental (such as an inadvertent release of documents) or intentional (such as a whistle-blower). Per unit time, each conspirator has some minuscule but non-zero chance of revealing the conspiracy, and this rate is denoted *p*. As our theory of this conspiracy process is memoryless, the exponential probability distribution is employed because, per any unit of time, revealing the conspiracy follows independent events at different moments over the time interval (i.e., a Poisson process). If the number of active conspirators as a function of time is *N*(*t*), then the hazard function is given by Φ(*t*) = 1 − (1 − *p*)^*N*(*t*)^. Thus it follows that the general form for the cumulative probability of conspiratorial failure at a time *t*, *L*(*t*), is

$$\begin{array}{c} L\left(t\right)=1-\text{exp}(-\int_{0}^{t}\Phi \left(t\right)dt). \end{array}$$ (1)

The precise form this will take depends on how the number of conspirators varies with time. The simplest case is when a conspiracy requires constant upkeep to maintain, and the number required to sustain the fiction is approximately constant with time. Typically this would describe some situation where active input to maintain the deception is vital. In such a case, *N*(*t*) = *N*_*o*_, a constant, and allowing *ψ* = 1 − *p* for brevity, the simple form is

$$\begin{array}{c} L_{c}\left(t\right)=1-\text{exp}(-t(1-\psi^{N_{o}})). \end{array}$$ (2)

**Fig 2:**
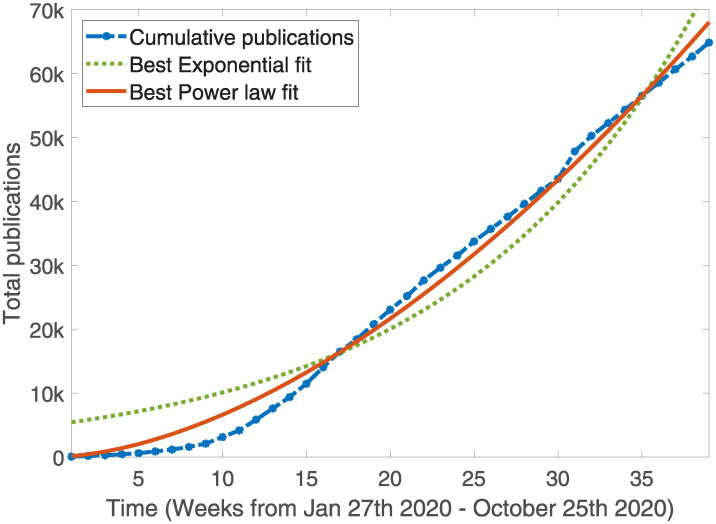
COVID related scientific publishing. Cumulative publishing is well-described by an empirical power law type function.

Previous work also looked at “single event” scenarios, where conspirators slowly died-off or were removed, presuming it could be established in the first instance. This is generally not appropriate for a hypothetical COVID conspiracy, which has occurred over a short time-frame and where increasing medical and scientific interest means that the number of complicit actors is growing rather than shrinking. One could argue that if COVID were an engineered bioweapon, then a “single event” conspiracy confined only to a small number of conspirators might in principle be possible. There are however several objections to this hypothetical framing, outlined in detail in the discussion section. With COVID marked by an explosion of scientific publishing, we require a conservative estimate of how the number of complicit agents changes with time. A conservative estimate of researchers involved can be garnered from cumulative COVID publications by week since the beginning of the pandemic, tracked by the LitCovid project [44]. As illustrated in Fig 2, publication growth obeys an approximately empirical power law, with the number of researchers with time given by *r*(*t*) = *r*_*o*_ *t*^*α*^, and *N*(*t*) = *N*_*o*_ + *r*(*t*). In this case, dynamic failure rate with time is given by

$$\begin{array}{c} L_{D}\left(t\right)=1-\text{exp}(-t-\psi^{N_{o}}(\frac{tE_{\frac{\alpha -1}{\alpha }}\left(-r_{o}t^{\alpha }\;\text{ln}\;\psi \right)}{\alpha }-\Gamma (1+\frac{1}{\alpha }){\left(-r_{o}\;\text{ln}\;\psi \right)}^{-\frac{1}{\alpha }})). \end{array}$$ (3)

where *E* is the Exponential integral function and Γ is the Euler gamma function.

### Parameter estimates

Table 1 gives essential parameters for simulation, and the rationale for selection. As with previous approaches, we can ascertain reasonable estimates of the number of complicit actors required to sustain a COVID conspiracy. For vaccination related conspiracy theory, we can estimate staff numbers in the relevant pharmaceutical companies, and for 5G conspiracy theories and related fringe ideas, we can garner a rough estimate of the approximately constant number of actors, *N*_*o*_, required to sustain various kinds of fictions. Non-exhaustive estimates for some of these figures are given in Table 2. It is important to note this list is far from complete, and only considers a selection of the actors required for an actual conspiracy. We deliberately underestimate the numbers to err on the side of caution, but these figures serve as illustration of the numbers required. As with prior approaches, we deliberately take estimates most favourable to conspirators to ascertain the best-case scenario under which they might endure. We can thus apply the model to deduce the likelihood of survival of different classes of COVID conspiracy, as outlined in the results section.

**Table 1:** Parameter estimates.

Parameter	Estimate	Comment
Failure probability per conspirator per unit time, *p*	7.69 × 10^−8^ per week	Taken from previous best-case estimates per conspirator for annual failure, scaled to weekly rate [42].
Initial number of research conspirators, *r*_*o*_	383 researchers	Fitting power law to publication data, with conservative underestimate of 3 novel complicit authors per paper [44].
Research growth rate, *α*	1.714	Taken from best-fit power law for publication growth (*R*^2^ = 0.9919) [44].

**Table 2:** Rough estimates of number of complicity actors required.

Public Health bodies (Selection)	
Organisation	Number Employed (Estimate)
World Health Organisation	7000
Centre for Disease Control (USA)	15000
Public Health England	5500
Robert Koch Institut (Germany)	1100
Korea Disease Control and Prevention Agency	1476
China Centre for Disease Control	2120
National Institute of Public Health (Japan)	4400
National Centre for Disease Control (India)	434
Federal Office of Public Health (Switzerland)	544
Finnish Institute for Health and Welfare	945
Instituto de Salud Carols III (Spain)	1164
Istituto Superiore di Sanit (Italy)	1808
National Institute for Public Health and the Environment (Netherlands)	1700
Public Health Agency of Sweden	450
Sciensaco (Belgium)	700
Sante Publqiue France	645
VECTOR (Russia)	1614
Public Health Agency of Canada	2379
National Sanitary Surveillence Agency (Brazil)	2206
European Centre for Disease Control	290
*Partial total (Public Health)*	***52,765***
**Vaccine / drug development (Selection)**	
Pfizer	116,500
AstraZeneca	57,500
Johnson and Johnson	122,200
Sanofi	105,000
GlaxoSmithKline	99,000
Janssen	40,000
Sinopharm	128,000
NovaVax	375
Sinovax	3000
Moderna	820
Gamaleya	379
Novartis	65,262
Roche	78,604
Merck	70,000
*Partial total (Pharmaceuticals)*	***886,640***
**Telecoms companies (Selection)**	
AT & T	248,000
Verizon	135,000
Nippon Telegraph	310,000
China Mobile	456,239
Deutsche Telekom	213,000
Softbank group Corp	74,952
China Telecom	281,215
Telefonica SA	113,819
Vodafone group	104,000
América Móvil	189,448
*Partial total (Telecommunications)*	***2,125,673***

Note that estimates in this list are far from exhaustive, and serve as a reasonable order of magnitude approximation of the minimal numbers required for complicity. Textual sources for these estimates are given in S1 File.

## Results

### A. Viability of COVID as a hoax / deliberately engineered conspiratorial narrative

**Fig 3:**
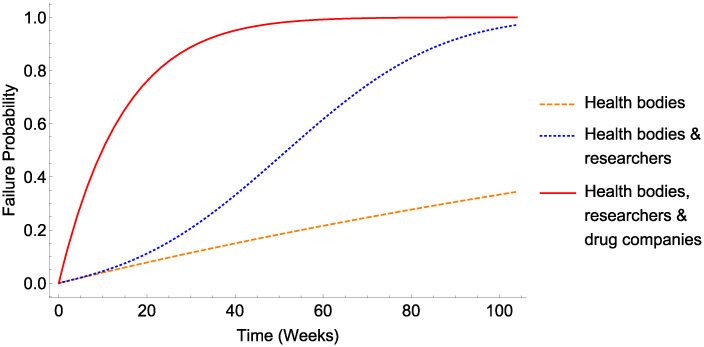
Viability of COVID hoax narratives. Viability of a COVID hoax with different classes of conspirator.

If COVID was a hoax or scam of some type, it follows that (a) public health bodies are complicit in this fiction or (b) that public health bodies and researchers are complicit in this deception or (c) public health bodies, researchers, and companies involved in vaccine and drug efforts are complicit in this hoax. If COVID were an engineered bioweapon, it would require at least the complicity of scientific researchers and public health bodies to maintain this fiction, given that the genomic sequence of COVID-19 has been publicly available since January 2020 (see discussion for further details). The likely endurance of these scenarios is shown in Fig 3.

### B. Viability of COVID-19 a pretext for a mass vaccination programme conspiratorial narrative

**Fig 4:**
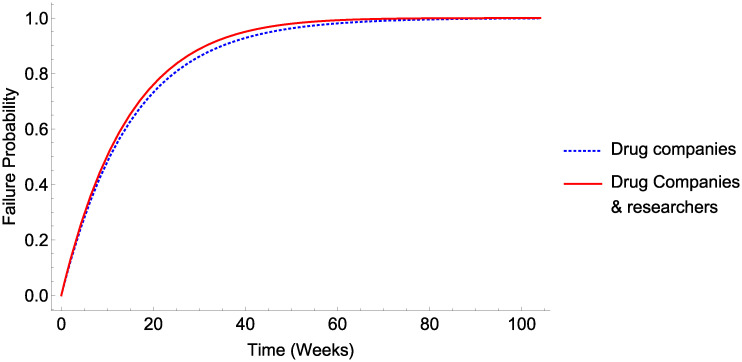
Viability of COVID vaccine plot narratives. Viability of a COVID hoax with different classes of conspirator.

Were COVID a scam or false justification for a vaccine, then either (a) drug companies alone or (b) drug companies and researchers would have to be complicit in the deception. The likelihood of these scenarios enduring are shown in Fig 4.

### C. Viability of a 5G Electromagnetic radiation-COVID link cover-up conspiratorial narrative

**Fig 5:**
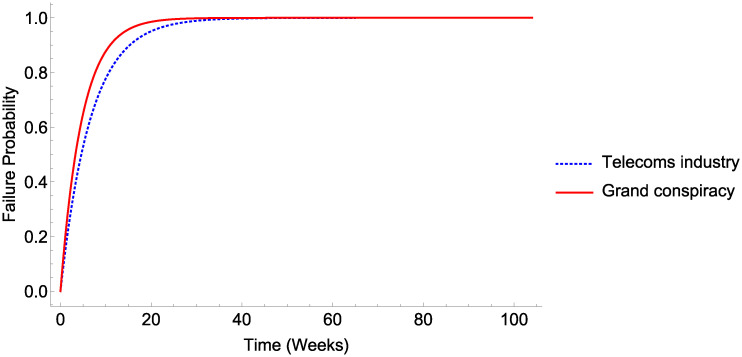
Viability of COVID-5G cover-up narratives. Viability of a COVID-5G cover-up with different classes of conspirator.

At the very least, a 5G link with COVID would require the complicity of the telecom industry, and potentially the addition of public health bodies, drug companies, and researchers too in a “grand conspiracy”. These scenarios are shown in Fig 5.

### Time to exposure of hypothetical COVID conspiracies

By manipulating Eqs 1–3, we can estimate to the nearest week how long it takes any hypothetical COVID conspiracy to have a given probability of exposure under different assumptions. This information is given in Table 3.

**Table 3:** Chances of exposure with time for different COVID conspiracies.

Conspirators	*L* ≥ 50%	*L* ≥ 75%	*L* ≥ 95%
Public health bodies only	171 weeks	342 weeks	740 weeks
Public health bodies / Researchers	50 weeks	71 weeks	97 weeks
Drug companies	11 weeks	21 weeks	45 weeks
Drug companies / Public Health bodies	10 weeks	20 weeks	43 weeks
Drug companies / Public Health bodies / Researchers	10 weeks	20 weeks	40 weeks
Telecoms companies	5 weeks	10 weeks	20 weeks
Grand conspiracy	4 weeks	7 weeks	14 weeks

## Discussion

As far back as 1517, the infamous Italian political philosopher Machiavelli advised leaders against engaging in conspiracy, observing that ‘many [conspiracies] have been revealed and crushed in their very beginning, and that if one has been kept secret among many men for a long time, it is held to be a miraculous thing’. Two centuries on, Benjamin Franklin observed pithily that ‘Three may keep a secret, if two of them are dead’ [45]. The results of this analysis give quantitative support to these assertions, and that depicted in Figs 3–5 inclusive demonstrate that even under circumstances most conducive to a hypothetical conspiracy, the long-term sustainability of these ostensible manipulations would tend to untenable.

If we were to take the smallest possible cohort, Public health bodies, then Table 3 suggests it might take over 3 years before the chances of the conspiracy being detected exceed 50%. Yet the simple and undeniable inclusion of researchers markedly decreases this time to under a year. When we consider that drug companies too are directly involved in COVID research efforts, then even only considering a selection of the actors involved drives the time to ≥ 50% exposure risk to a mere 10 weeks. The sheer size of just the largest telecoms companies also makes 5G-COVID narratives unviable—even if only the Telecoms companies were complicit, such a narrative would be more likely than not to fall apart by 5 weeks. If, as is more likely, all these parties would be required to sustain a fiction, the time to likely detection would decrease to under a month.

Even under ideal circumstances for conspirators, our Devil’s advocate approach does not seem to support the hyperbolic claims frequently shared online about COVID. It is also worth emphasising that parameter estimates used in this work were deliberately selected to be maximally conducive to conspirators. The value of *p* in particular, the intrinsic failure rate per conspirator per unit time, was the lowest realistic value achievable. In previous work, the highest values for *p* were approximately two orders of magnitude above this [42]. The number of actors included in Table 2 represent an incredibly conservative estimate too, and a more comprehensive quantification would unavoidably drive up the failure rate for all COVID conspiracies.

It is worthwhile too to address one potentially derailing criticism—one might object that simply including all member of an organisation as complicit actors in a conspiracy is too simplistic. For example, it is possible to argue that a conspiracy is so compartmentalised that only certain members would be aware of its existence, even inside complicit bodies. This compartmentalisation argument is much beloved of conspiracy theorists, but it fails utterly in a hypothetical medico-scientific conspiracy, because falsification and investigation are cornerstones of scientific research. We do not simply accept a claim as true without external review and validation, and independent investigation.

There are a few potential objections to this that must be broached, however. In relation to the claims that COVID might be a bioweapon, it would be argued that this is a “single-event” conspiracy requiring only a small number of actors. But if this were the case, such a manipulation would have to be extremely subtle, as the COVID genome was sequenced and publicly available by January 10th 2020, and betrays no evidence of human tampering. If evidence of anthropogenic manipulation existed, then subsequent researchers and health bodies would have to endeavour to keep it suppressed rather than just the hypothetical initial cohort. Alternatively, one could argue that COVID is man-made and yet is indistinguishable from anything naturally occurring. This assertion borders on the non-falsifiable, and inverts the burden of proof. Claims that pandemics are human-crafted weapons are centuries old. Yet even in the modern era, biological weapons are impractical, being nigh on impossible to aim. This renders a hypothetical infectious bioweapon as dangerous to its makers as its intended target. Accordingly, we do not treat a COVID bioweapon conspiracy as a single-event in this work.

There are other concerns too—while science is in principle self-correcting, some trepidation about this process is understandable. The influence of industry raise concerns about conflicts of interest—one might consider the machinations of the tobacco industry to undermine the scientific evidence that smoking was carcinogenic, or similar efforts by the fossil fuel lobby to sty-my action on climate-change. But it is worth appreciating that in these prominent examples, it was scientific and medical investigation which exposed odious effects [45]. Far from buttress conspiratorial claims, these examples showcase that scientific investigation can derail even vested interests, despite attempts at false balance [46] by those industries. More subtle and perhaps more troubling however is the reality that science itself is far from perfect, and much published biomedical research is non-replicable or otherwise suspect [47, 48]. Yet while this a substantial problem, the bias in scientific publishing leans towards “positive” findings over the null result [49]. Thus, researchers who could show a link between COVID and 5G would be more likely to get that finding published than those who merely demonstrated the null hypothesis of no effect, even if that ostensibly positive finding was in reality a false positive. For all the myriad flaws in science, it does not make conspiracy more likely or sustainable.

**Fig 6:**
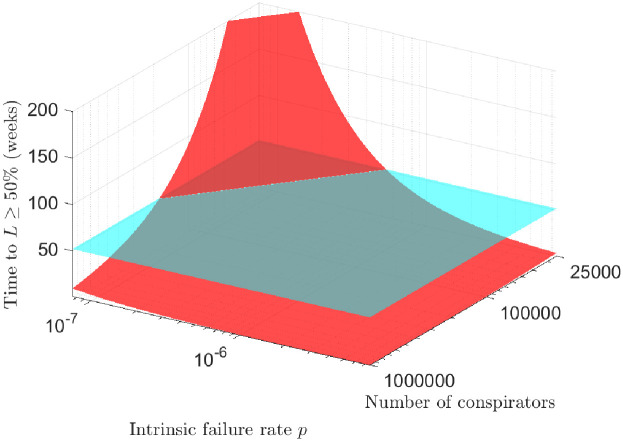
Durability of conspiratorial narratives with variation of *p* and number of conspirators. The light-coloured plane denotes 52 weeks (1 year) for visual clarity.

Accordingly, for a medico-scientific conspiracy to flourish, it would require all medical and scientific experts involved to become complicit in the narrative (or grossly and systematically incompetent) and all health bodies to be equally nefarious. Even if exact numbers of agents required are unknowable to any high degree of precision, we can perform a robustness analysis, varying *p* by two orders of magnitude and the number of conspirators varying from 25, 000 to 1 million. Using the simplest form given in Eq 1 which is unrealistically generous to conspirators, Fig 6 depicts the time until likely detection of the conspiracy. As might be intuitively guessed, a conspiracy can only hold for appreciable time if both *p* is small and the number of conspirators is limited—neither situation likely applicable to COVID-19 narratives.

It is also important to point out that this simple model does not consider extrinsic factors, and only pivots on the assumption that a conspiracy is revealed from within, either deliberately or inadvertently. There would also of course be extrinsic factors, such as third parties who could expose a clandestine plot, but they are not considered here. Their influence would be to increase the likelihood of a conspiratorial failure, and again the situation depicted here is the best-case scenario for hypothetical conspiracy theorists. A more comprehensive model also considering this could be useful in future, but is not considered here for brevity.

While it is hoped the discussion and analysis presented here might prove useful in disabusing certain individuals of misguided beliefs, it is important to note that all strategies have limitations. It is also worthwhile to consider that ideological motivations frequently underpin our acceptance of different narratives, and these narratives themselves are frequently intrinsically politicised. Acceptance of the scientific consensus on climate change, for example, correlates strongly with one’s political leanings, and those with pronounced free-market views are far more likely to tend towards rejection of this reality [45, 50–52]. Research by Kahan and colleagues has demonstrated that political and ideological positions can lead individuals to distort objective information to buttress their worldview, on everything from gun control [53] to the HPV vaccine [54]. While beyond the scope of this work, it might be worthwhile to investigate whether the informal observation that conservative outlets appeared to amplify “COVID is a hoax” narratives might be a manifestation of this phenomena.

The dominance of disinformation is a product of both organic growth of long-established conspiracy theories, and deliberate disinformation. It is worth noting that political disinformation on medical science has a long and ignoble history, and sometimes long-standing consequences. Similar to the current claims that COVID is a bio-weapon, a 1980s soviet disinformation campaign entitled Operation INFEKTION propagated the myth that AIDs was man-made. To this day, the myth retains significant traction in American communities significantly affected [45]. Before COVID-19 was even recognised, Russian state forces also perpetuated myths about the dangers of 5G [55]. The role of social media in the propagation of contemporary mistruth has been significant too, with this author arguing previously that they shoulder much of the responsibly for the ubiquity of dangerous fictions [56]. It also cannot be ignored that amplification of falsehoods during the pandemic by celebrities, politicians, and ‘elites’ has been unavoidable. To take but one example, Donald Trump repeatedly spread inaccurate information about the virus and treatments for it while still American president [57, 58]. Such statements and celebrity coverage are likely to skew public perception [59]. How much precisely various factors contribute to amplification of falsehoods is beyond the scope of this work, but remains a pertinent and important question.

Deeply-seated conspiratorial beliefs are seemingly impervious to intrusions of evidence and reality. Much of the virulent disinformation about COVID has a distinct anti-authoritarian drive, and contempt for expertise. This is not unprecedented, with researching that vocal conspiracy theorists pride themselves on being too special to be duped [60]. This does not appear to be exclusively a function of function of education or political leaning; one French study found COVID anti-mask protesters across the political spectrum, consisting primarily of women with higher education. While coming from disparate groups, these individuals were unified by a perception of themselves ‘free-thinkers’, rejecting perceived authority [61].

Such refrains are unfortunately common in conspiratorial circles, with psychological studies consistently show a significant proportion motivated by an egotistical drive, and feeling of authority it induces [23, 60, 62]. With COVID-19, there is evidence that acceptance of conspiracy theory on the topic stems in part from a psychological disposition to reject information coming from experts and other authority figures [63]. Frequently this confidence in their beliefs is inversely proportional to their actual understanding. In one especially glaring example, anti-vaccine activists who proclaimed to know the most about vaccination and autism actually scored lowest in their knowledge of both subjects, despite rating their understanding as high [64]—a potent example of the Dunning-Kruger phenomenon [65], the observation that those least competent drastically overrate their understanding and ability. In many instances, the mere conviction that conspiracy theorists know more than others is especially intoxicating, and this motivation can be nigh on impossible to address [45].

Even with this in mind, however, we must remember acceptance of medico-scientific consensus is a spectrum (Fig 1) and such individuals constitute only the most extreme fringe. Many who harbour fears and suspicions hover more central on that continuum, and eminently reachable. Rather than dismiss concerns, we must view people rendered uncertain by these claims as victims of conspiracy theorists, vulnerable to the disinformation perpetuated by others. It is critical that the scientific and medical community learn how to better address this; it is hoped that the methodology in this work can help towards assuaging some fears and doubt those subjected to ostensibly convincing conspiratorial narratives may experience, serving as riposte to common misguided narratives.

Ultimately however, a serious conversation about how we address the dominance of medico-scientific conspiracy theories is urgently required. The COVID-19 crisis has laid bare the weaknesses in our system, and our inability to respond robustly to disinformation. Emerging evidence suggests that we can be immunised against certain forms of falsehood, provided this intervention comes before exposure [66]. Such an endeavour demands we embrace information hygiene as a society [56], encouraging people to treat all information as potentially pathogenic before they accept or propagate it. The potentially negative influence of social media companies on public understanding of science and medicine demands immediate investigation and further research too [56]. In the interim, it is vital that physicians and scientists begin to address the odious influence of disinformation, before it undermines the vast strides we have made in the centuries since the enlightenment. Our future well-being is dependent upon it.

## Supporting information

S1 File Source estimates. Sources for estimates in Table 2.
(XLSX)
